# Exploring Construction of Biomedical Ti6Al4V-Ti5Cu Composite Alloy with Interpenetrating Structure: Microstructure and Corrosion Resistance

**DOI:** 10.3390/ma18030491

**Published:** 2025-01-22

**Authors:** Yuan Zhou, Qing Zhao, Ruchen Hong, Dongyi Mai, Yanjin Lu, Jinxin Lin

**Affiliations:** 1 College of Chemistry, Fuzhou University, Fuzhou 350108, China; zhouyuan@fjirsm.ac.cn (Y.Z.); zhaoqing@fjirsm.ac.cn (Q.Z.); hongruchen@fjirsm.ac.cn (R.H.); 2 Key Laboratory of Optoelectronic Materials Chemistry and Physics, Fujian Institute of Research on the Structure of Matter, Chinese Academy of Sciences, Fuzhou 350002, China; maidongyi@fjirsm.ac.cn; 3 Key Laboratory of Opto-Electronic Science and Technology for Medicine of Ministry of Education, College of Photonic and Electronic Engineering, Fujian Normal University, Fuzhou 350117, China; 4 Fujian Science & Technology Innovation Laboratory for Optoelectronic Information of China, Fuzhou 350108, China

**Keywords:** corrosion, microstructures, powder processing, interface

## Abstract

Cu-bearing titanium alloys exhibit promising antibacterial properties for clinical use. A novel Ti6Al4V-Ti5Cu composite alloy is developed using powder bed fusion (selective laser sintering, SLM) and spark plasma sintering (SPS). SLM produces a triple periodic minimal surface (TPMS) lattice structure from Ti6Al4V, which is then filled with Ti-5Cu powders and sintered using SPS. Microstructural analysis confirms a well-bonded interface between Ti6Al4V and Ti-5Cu could be achieved through SLM-SPS technology. The composite primarily showcases laths α phase, with Ti_2_Cu precipitates in the Ti-5Cu region. Electrochemical assessments reveal superior corrosion resistance in the Ti6Al4V-Ti5Cu composite compared to SLM-Ti6Al4V and SPS-Ti-5Cu. The antibacterial rate of the TPMS structure exceeds 90%, and that of TCCU-90 reaches as high as 99%, manifesting robust antibacterial activity. These findings suggest a strategy for creating biomimetic alloys that seamlessly combine structure and multifunctionality within biomedical materials.

## 1. Introduction

Pure titanium (CP-Ti) and Ti6Al4V alloys are widely used in dental implants and joint prostheses due to their excellent mechanical properties, biocompatibility, and corrosion resistance [1,2,3]. According to ASTM F67 [4], implants are required to have good compressive properties, elastic modulus, and wear resistance; compared with stainless steel (316L) and Cobalt-based alloys, titanium alloys have better biocompatibility and are closer to the elastic modulus of human bone [5]. However, implant-induced bacterial infection remains a critical concern leading to implant failure [6,7,8,9]. In response, antibacterial titanium alloys containing Cu, Zn, and Ag elements, such as Ti6Al4V-5Cu [10], Ti-10Cu [11], and Ti-3Ag [12] alloys, have been developed to combat this issue. Research by Liu et al. [10] highlighted the potent bactericidal properties of Ti6Al4V-5Cu alloys against *Escherichia coli*, exhibiting a high antibacterial rate of 98%. Furthermore, research indicated that titanium alloys containing more than 5 wt.% copper can significantly decrease bone resorption and enhance the formation of new bone [11]. The antibacterial efficacy of titanium alloys with copper is closely linked to the copper concentration. Typically, when the Cu content exceeds 5 wt.% in a titanium alloy, it can achieve an antibacterial effectiveness of around 98% against *Escherichia coli* and *Staphylococcus aureus* [10,13,14,15].

Drawing inspiration from natural biomaterial structures can serve as a blueprint for designing metallic materials that seamlessly integrate structure and function. Bioinspired structural architectures offer an effective strategy towards this goal. For instance, Ma et al. [14] developed Mg-Ti biomimetic composites via a two-step approach involving selective laser melting (SLM) and pressureless infiltration. In this process, a porous Ti6Al4V scaffold (acting as the hard phase) featured a mutually interpenetrating and continuous structure, providing a three-dimensional framework into which pure Mg melt (the soft phase) was integrated. This architectural arrangement facilitated stress distribution, increased crack propagation resistance, and significantly enhanced strength, ductility, fracture toughness, and impact resistance. However, this method had limitations as it required distinct components with significantly different melting points, restricting its universal application for metallic materials with similar melting points. To address this challenge, we recently proposed a modified two-step technique utilizing SLM and hot isostatic pressing (HIP) to produce a nacre-like Ti6Al4V-CPTi composite alloy [16,17]. Here, SLM-produced Ti6Al4V served as the mortar, while CPTi bonded with Ti6Al4V via HIP served as the brick. The composite produced displayed a distinctive blend of high strength, excellent fracture toughness, and notable corrosion resistance. However, it is important to note that HIP processing involves relatively high costs, which limits its broader application for metallic composites with bionic structures. Spark plasma sintering (SPS) technology harnesses the electric spark discharge effect to create intense heat and pressure among metal powders, kick-starting their sintering process. In the SPS procedure, pulsed currents with high energy and low pressure rapidly elevate temperatures between adjacent particles, leading to spark plasma discharge. This discharge event produces thermal energy, ionized diffusion, and a phenomenon known as “pulse pressure”, all of which synergistically enhance atomic diffusion at the contact points, facilitating the sintering of the powders. SPS enables rapid powder consolidation by applying pulsed current and axial pressure simultaneously at approximately 60% of the materials’ melting point [18,19]. SPS has shown promise in producing a dense CP-Ti and Ti6Al4V alloy at temperatures below 850 °C with a pressure of 50 MPa [20], while the dense Ti6Al4V alloy can be obtained at a sintering temperature below 850 °C [21,22]. There is growing interest in applying SPS to produce various titanium alloys with a fine structure to achieve superior mechanical properties. Numerous studies have confirmed that dense titanium alloys can be obtained at sintering temperatures of 750–950 °C under a 50 MPa pressure during the SPS process [23]. For instance, Long et al. [22], who fabricated Ti6Al4V using SPS at 850 °C for 4 min under a pressure of 50 MPa, reported that the resulting SPS-Ti6Al4V alloy exhibited an ultrafine-grained microstructure consisting of α and β phases, with a size range of 0.51–0.89 μm. Hence, SPS technology emerges as a potential candidate to replace the HIP process in fabricating bioinspired structural titanium composites, offering a more cost-effective alternative. In this study, our aim is to explore an alternative approach that combines SLM with spark plasma sintering (SPS) technology for fabricating Cu-bearing titanium composite alloys.

In this study, we embarked on fabricating an innovative Ti6Al4V-Ti5Cu composite alloy using a combination of SLM and SPS techniques, aiming to endow titanium alloy with both structural integrity and antibacterial functionality. Initially, we utilized SLM to create Ti6Al4V triple periodic minimal surface (TPMS) scaffolds with a sheet-gyroid unit featuring diverse pore sizes. Subsequently, these scaffolds were filled with Ti-5Cu powders and underwent sintering using SPS technology. The TPMS framework allows for the generation of various porous structures by adjusting and filling curved surfaces, offering a method for designing and producing parametric porous scaffolds with intricate functional gradients and excellent permeability [24]. Consequently, the sheet-gyroid TPMS scaffold produced by SLM in Ti6Al4V powders, acting as the hard phase, was chosen as a constituent, establishing a three-dimensional architecture alongside the Ti-5Cu component, serving as the soft phase. This configuration mirrors the mutually interpenetrating and continuous arrangement seen in natural biomaterials. The objective is to assess the corrosion resistance and antibacterial properties of the resulting Ti6Al4V-Ti5Cu composite. The anticipated outcome of this research is to introduce a novel concept for medical-grade titanium alloys that combines structural intricacy with multifunctionality.

## 2. Materials and Methods

### 2.1. Materials Preparation

Sheet-gyroid, also referred to as the double gyroid or gyroid foam due to both wall sides representing gyroid surfaces, served as the framework for filling Ti-5Cu powder. These sheet-gyroid TPMS scaffolds, possessing porosity levels of 70%, 80%, and 90%, were generated via computer-aided design software (Wolfram Mathematica 12) using Equation (1):

(1) $$\varphi_{Sk-G}\left(x,\;y,z\right)=\sin\left(ax\right)\cos\left(ax\right)+\sin\left(ay\right)\cos\left(az\right)+\sin\left(az\right)\cos\left(ax\right)=C$$

Equation (1) defines the sheet-gyroid TPMS scaffold, denoted as $\varphi_{Sk-G}$(x, y, z), as an implicit function involving x, y, and z. Here, C controls the matrix phase’s width, determining gyroid structure porosity, while ’a’ governs the TPMS surface’s periodicity. Porosity (P) of the sheet-gyroid unit cell structures was regulated by Equation (2), where vs represents solid unit volume, and V_0_ denotes periodic cube volume. The detailed design and porosity regulation could be found in our previous study [24].

(2) $$P=\left(1-\frac{V_{S}}{V_{0}}\right)\times 100\%$$

**Figure 1:**
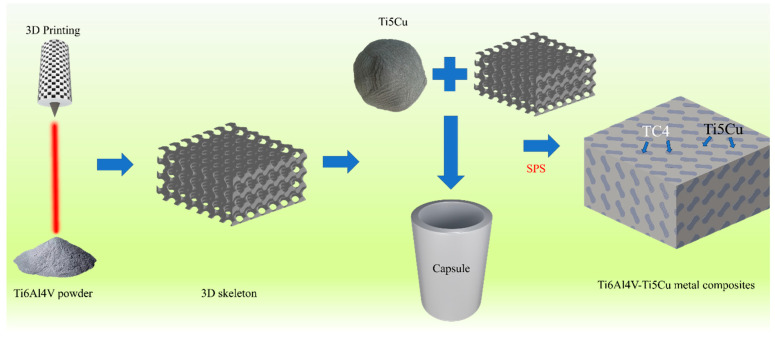
Producing Ti6Al4V-Ti5Cu composite by a two-step approach consisting of SLM and SPS.

**Figure 2:**
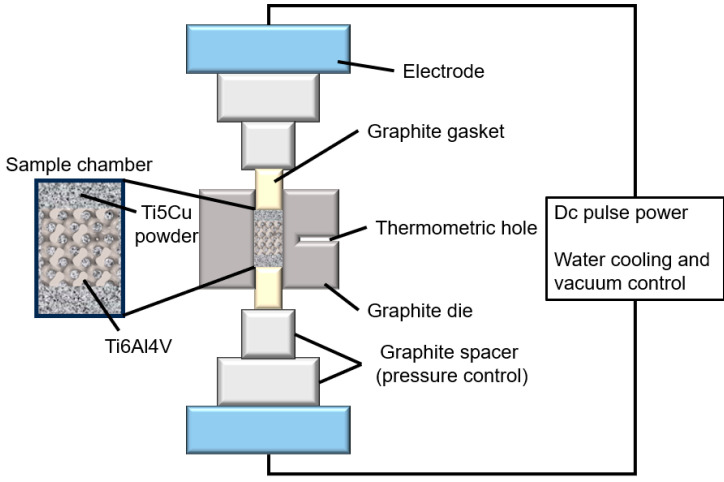
SPS equipment diagram.

Conceptlaser’s Mlab-R SLM device is used to prepare the TPMS porous scaffold. The laser power is 95 W, the scanning speed is 900 mm/s, the thickness is 25 μm, and the laser track width is 0.11 mm. Post-production, Ti-5Cu powders (high-purity titanium powder and 5 wt.% high-purity copper powder were ball milled for 1 h) were filled and compacted into the sheet-gyroid scaffolds. The next stage consisted of sintering the Ti-5Cu powder mixture to form a Ti6Al4V-Ti5Cu composite. This sintering procedure was conducted through spark plasma sintering (SPS) at 920 °C, with a pressure of 50 MPa, heating up at 100 °C/min, and held for 5 min. The schematic diagram of manufacturing the Ti6Al4V-Ti5Cu composite by a two-step approach consisting of SLM and SPS is shown in Figure 1. SPS sintering equipment is shown in Figure 2.

### 2.2. Materials Analysis

Prior to microstructure analysis, all samples were ground with 2000# grit SiC sandpaper. Subsequently, each sample was polished with 1 μm diamond paste. After this, the sample was subjected to etching in a Kroll reagent consisting of 6 mL HF, 10 mL HNO_3_, and 50 mL H_2_O. The microstructure was examined using a Helios G4 CX scanning electron microscope (SEM) equipped with energy dispersive spectroscopy (EDS) from ThermoFisher (Waltham, MA, USA). The alloy’s phase composition was assessed through Cu Kα radiation X-ray diffraction (XRD) using the D/MAX-2500PC instrument by Rigaku, Tokyo, Japan.

### 2.3. Electrochemical Test

**Figure 3:**
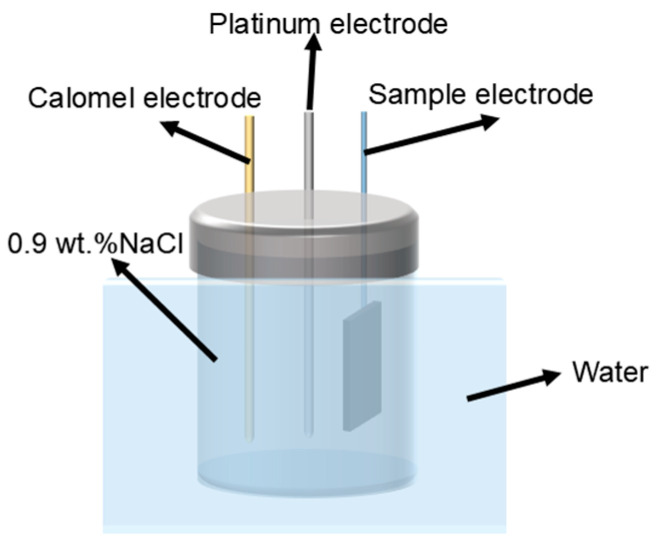
Electrochemical test set.

The electrochemical experiment was carried out by reference 600+ electrochemical workstation developed by Gamry Company in Philadelphia, PA, USA, with three samples tested per group. Based on ISO 16429:2004 [25], open circuit potential (OCP), potentiodynamic polarization, and electrochemical impedance spectroscopy (EIS) measurements were performed in 0.9 wt.% NaCl solution in a standard three-electrode setup, using a water bath at a test temperature of 37 ± 1 °C. The schematic diagram of the test device is shown in the Figure 3, in which the calomel electrode is the reference electrode, the platinum electrode is the opposite electrode, and the sample electrode is connected to the working electrode. The OCP test ran for 1 h to achieve a stable potential value. Subsequently, electrochemical impedance spectroscopy was conducted over a frequency range of 10^2^~10^5^ Hz and an amplitude range of 10 mV. The potentiodynamic polarization scanning rate was set at 0.025 V/s, with a scanning voltage range of 0.5~2.0 V. The electrochemical test data were analyzed using Zview 3.3 and Cview 3.5 software. Corrosion potential and corrosion current density were determined through Tafel fitting. Ti6Al4V-Ti5Cu composite samples were immersed in a 0.9 wt.% NaCl solution at 37 °C for 7 days to evaluate corrosion behavior. The surface morphology was examined using the Agilent 5500 atomic force microscope)AFM, manufactured by Agilent Corporation of Santa Clara, CA, USA.

### 2.4. Antibacterial Experiment

In this research, the Gram-negative bacterium *Escherichia coli* (*E. coli*) was employed. The antibacterial assessment was conducted following the plate counting method outlined in Chinese standard GB/T 2591-2003 [26]. Each sample had dimensions of 10 mm × 10 mm × 2 mm, was polished with 2000# sandpaper, and sterilized at 121 °C for 30 min. The initial concentration of *E. coli* suspension was 1 × 10^8^ cfu/mL, which was then diluted to 1 × 10^5^ cfu/mL using PBS solution. The samples were placed in a 24-well culture plate, and 50 μL of the diluted bacterial suspension was added to each well. The plate was then placed in an incubator at 37 °C for 24 h at 90% relative humidity. Subsequently, 2 mL of normal saline solution was utilized to rinse off the bacteria from the sample surfaces. The liquid from the 50 μL wells was evenly spread on agar plates, which were then cultured at 37 °C for another 24 h. Following the 24-h co-culture period, images of the colonies were captured, and the antibacterial efficiency was calculated according to Equation (3):

(3) $$Antibacterial\;rate\left(R,\%\right)=\frac{N_{control}-N_{experiment}}{N_{control}}\times 100\%$$

where N_control_ represents the average number of bacterial colonies in the control sample (Ti6Al4V), and N_experiment_ is the TCCU alloy. Three parallel samples were prepared for each experiment and were repeated three times.

## 3. Results

### 3.1. Microstructural Observation

**Figure 4:**
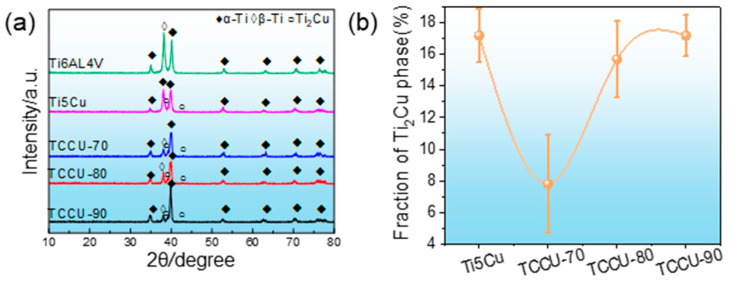
(**a**) XRD pattern and (**b**) the fraction of Ti_2_Cu phase in matrix of the Ti-5Cu, TCCU-70, TCCU-80, and TCCU-90 alloys.

The Ti6Al4V, Ti-5Cu, TCCU-70, TCCU-80, and TCCU-90 alloys exhibited relative densities of 99.76%, 99.64%, 99.60%, 99.66%, and 99.684%, respectively, signifying the successful production of highly-densified samples. As depicted in Figure 4a, XRD patterns of these alloys revealed the dominance of the α phase in their matrices, while the presence of the Ti_2_Cu phase was detected in Ti-5Cu, TCCU-70, TCCU-80, and TCCU-90 alloys. Analysis of the Ti_2_Cu phase content, as shown in Figure 4b, indicated fractions of 17.22%, 7.80%, 15.75%, and 17.05% in Ti-5Cu, TCCU-70, TCCU-80, and TCCU-90, respectively. This underscores a significant correlation between the Ti_2_Cu fraction and the TPMS porosity. However, no discernible peaks attributed to the β phase were detected.

**Figure 5:**
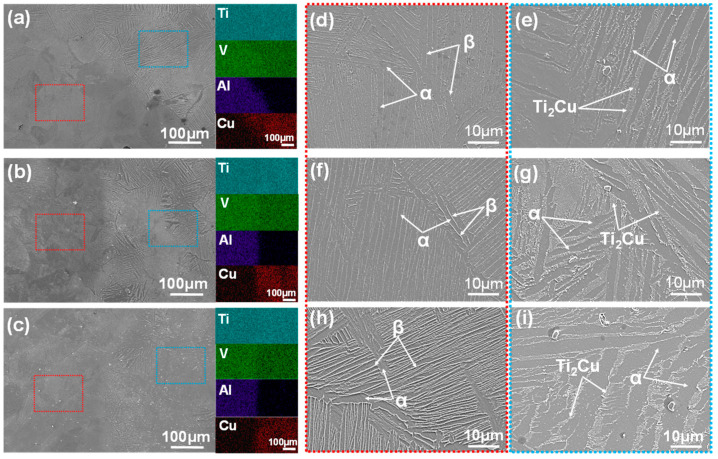
The SEM microstructure and the corresponding EDS maps of the (**a**) TCCU-70, (**b**) TCCU-80, and (**c**) TCCU-90 alloys; the microstructure at high magnification of the Ti6Al4V-zone marked by the red dotted line: (**d**) TCCU-70, (**f**) TCCU-80, and (**h**) TCCU-90 alloys; the microstructure at high magnification of the Ti-5Cu-zone marked by the blue dotted line: (**e**) TCCU-70, (**g**) TCCU-80, and (**i**) TCCU-90 alloys.

The SEM micrographs and accompanying EDS maps depicted in Figure 5a–c illustrate the microstructures of the TCCU-70, TCCU-80, and TCCU-90 alloys. EDS mapping confirmed the absence of defects like pores, micro-cracks, or precipitates along the interface between Ti-5Cu and the Ti6Al4V scaffold, signifying a robust metallurgical bond achieved through SPS processing. The EDS maps also highlighted the uniform distribution of Ti and V elements in the matrix, while Al and Cu elements were notably enriched in the Ti6Al4V and Ti-5Cu zones, respectively. The distribution pattern of the V element from the Ti6Al4V zone to the Ti-5Cu zone could be attributed to its lower diffusion activation energy Q and higher diffusion speed within the temperature range of 1173 to 1273 K, as explained in our prior research [16,17]. Furthermore, when observing the microstructures at higher magnification, specifically in Figure 5d–h and Figure 5e–i, the microstructure within the Ti6Al4V and Ti-5Cu zones was predominantly composed of the α and β laminar phase, with Ti_2_Cu precipitates dispersed in the Ti-5Cu zone. Notably, an increase in porosity induced the formation of Ti_2_Cu precipitates within the Ti-5Cu zone, consistent with the XRD findings.

**Figure 6:**
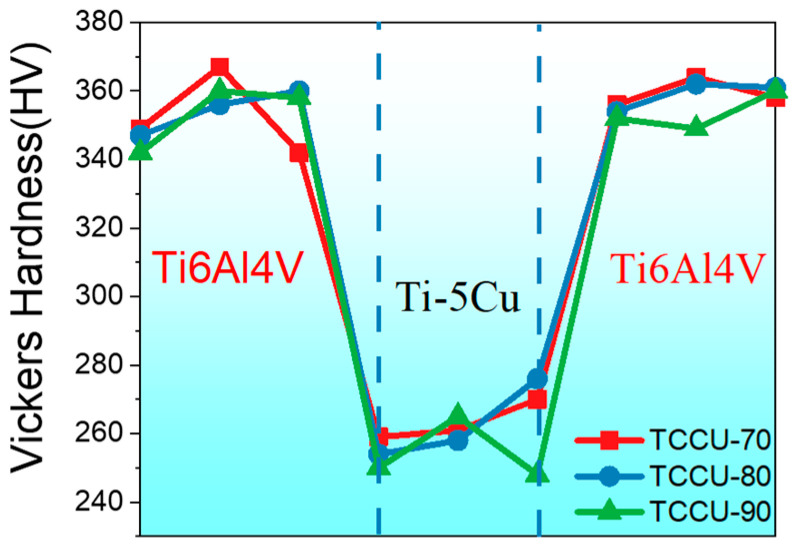
Microhardness distribution across the interface between the Ti6Al4V and Ti5Cu zone.

Figure 6 illustrates the microhardness distribution along the interface between Ti6Al4V and Ti5Cu. Generally, the Ti6Al4V zone, formed via SLM, consistently displayed a microhardness around 350 Hv, whereas the Ti5Cu zone showed a reduced value of approximately 260 Hv. This pattern suggests that the Ti6Al4V zone functioned as a harder phase, while the Ti5Cu served as a softer phase in the bioinspired composite alloys.

### 3.2. Corrosion Behavior

**Figure 7:**
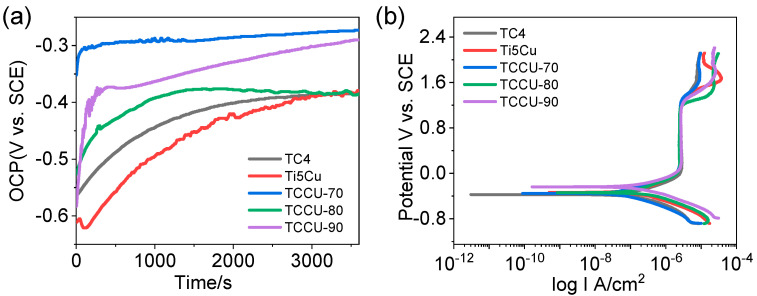
(**a**) Open circuit potential curves, (**b**) potentiodynamic polarization curves of the Ti6Al4V, Ti-5Cu, TCCU-70, TCCU-80, and TCCU-90 alloys.

To assess the corrosion properties of interpenetrated titanium composite alloys, electrochemical tests were conducted on TCCU-70, TCCU-80, and TCCU-90 alloys. The open circuit potential (OCP) curves, shown in Figure 7a, indicate an increasing trend in positive values when immersed in a 0.9 wt.% NaCl solution at 37 °C, suggesting the formation of a passive film on the surface. After a 3600 s evaluation, the OCP values were ranked as follows: TCCU-80 > TCCU-90 > Ti6Al4V > Ti-5Cu > TCCU-70. The TCCU-80 and TCCU-90 alloys demonstrated notably higher OCP values, whereas no significant differences were observed among Ti6Al4V, Ti-5Cu, and TCCU-70. Elevated OCP values typically indicate slower anodic reaction kinetics, which corresponds to lower corrosion susceptibility. However, from a thermodynamic perspective, OCP alone cannot conclusively determine the corrosion rate of metallic materials. Therefore, further evaluation of corrosion resistance was carried out using potentiodynamic polarization tests and electrochemical impedance spectroscopy.

Figure 7b illustrates the polarization curves for Ti6Al4V, Ti-5Cu, TCCU-70, TCCU-80, and TCCU-90 alloys in a 0.9 wt.% NaCl solution at 37 °C, with relevant corrosion parameters detailed in Table 1. All curves exhibit a similar pattern consisting of Tafel, passivation, and transpassive regions, indicating similar corrosion behavior across all samples. A passivation zone is evident where the current density remains nearly constant before reaching the breakdown potential, indicating good passivation ability for the titanium composite alloys, similar to Ti6Al4V and Ti-5Cu alloys. In addition, the passivation zone of the composite is similar to that of Ti6Al4V and Ti-5Cu alloys, indicating that a stable passivation film is easily formed on the surface of the composite. Furthermore, it is evident from the polarization curves that introducing interfaces also caused a positive shift in corrosion potential. The positive shift of corrosion potential reflects the increasing trend of corrosion resistance of the alloy. The E_corr_ values for Ti6Al4V, Ti-5Cu, TCCU-70, TCCU-80, and TCCU-90 alloys were −0.31 ± 0.02 V, −0.34 ± 0.01 V, −0.38 ± 0.03 V, −0.38 ± 0.02 V, and −0.28 ± 0.04 V, respectively. Interestingly, increasing the porosity of the TMPS scaffold appeared to increase the corrosion potential, indicating that this interpenetrated structure could enhance the corrosion thermodynamics of Ti6Al4V and Ti-5Cu. The I_corr_ values for Ti6Al4V, Ti-5Cu, TCCU-70, TCCU-80, and TCCU-90 alloys were (2.41 ± 0.21) × 10^−7^ A·cm^−2^, (1.93 ± 0.37) × 10^−7^ A·cm^−2^, (1.58 ± 0.74) × 10^−7^A·cm^−2^, (1.69 ± 0.38) × 10^−7^ A·cm^−2^, and (1.83 ± 0.63) × 10^−7^A·cm^−2^, respectively. The lower the I_corr_, the stronger the corrosion resistance of the alloy. Clearly, Ti-5Cu exhibited the highest corrosion current density compared to the other groups. Conversely, this situation changed after Ti-5Cu was metallurgically arranged with Ti6Al4V in the interpenetrated TMPS structure. Generally, the corrosion current (I_corr_) value of the composite titanium alloy was found to be lower than that of Ti6Al4V and Ti-5Cu. This suggests that the porosity of the TMPS structure did not affect the corrosion resistance of the composites. The lower corrosion rate observed in the Ti6Al4V-Ti5Cu composites supported this, providing additional evidence of their corrosion resistance.

**Figure 8:**
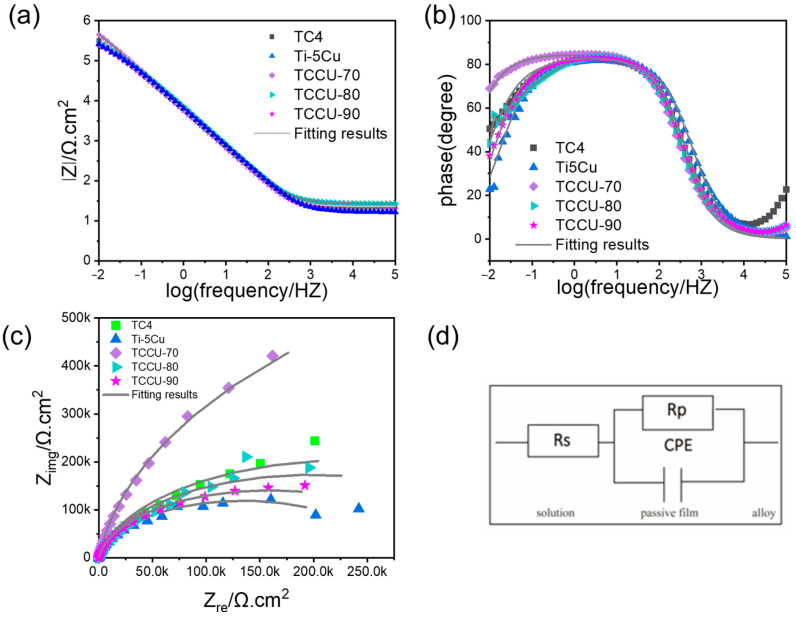
(**a**) Nyquist diagrams, (**b**) Bode-impedance modulus, and (**c**) Bode-phase plots of the Ti6Al4V, Ti-5Cu, TCCU-70, TCCU-80, and TCCU-90 alloys; (**d**) equivalent electrical circuit.

The electrochemical impedance spectroscopy (EIS) results for Ti6Al4V, Ti-5Cu, TCCU-70, TCCU-80, and TCCU-90 alloys, measured in a 0.9 wt.% NaCl solution at 37 °C, are shown in Figure 8. The Nyquist plots in Figure 8a reveal capacitive semicircles for all alloys, indicating similar corrosion mechanisms. Generally, a larger capacitive semicircle implies better corrosion resistance. The TCCU-70 alloy displayed the largest semicircle diameter in Figure 8a, suggesting it has the highest corrosion resistance among the tested alloys.

Additionally, Figure 8b and Figure 8c presents the Bode-impedance modulus and Bode-phase plots, respectively. The Bode-impedance modulus in Figure 8b shows a linear slope of about −1 across the frequency range of 10^−2^ to 10^3^ Hz, which signifies good capacitances for all samples. The |Z| modulus values increased in the following order: TCCU-90 < Ti-5Cu < Ti6Al4V < TCCU-80 < TCCU-70, with TCCU-70 exhibiting the highest impedance modulus |Z|. This suggests that the passive film on the TCCU-70 alloy offers superior corrosion protection against the electrolyte. Regarding the Bode-phase data in Figure 8c, the maximum phase angle for all samples remained around ~85° within the frequency range of 10^−2^ to 10^2^ Hz. This suggests that the passive films on all samples effectively inhibit the penetration of the electrolyte into the matrix.

To assess the electrochemical properties of the passive film, an equivalent circuit (EEC) model was utilized for analyzing the EIS data, as depicted in Figure 8d. In this EEC model, the constant phase element (CPE) is arranged in parallel with the oxide film resistor (R_p_), while the solution resistor (R_s_) is configured in series with the first two components. Due to the frequency-dependent nature of capacitance, CPE is utilized within the EEC framework. Given the complexity of corrosion processes at the interface, the ideal capacitive element has been substituted with the CPE, and the impedance of the CPE is described by Equation (4), as follows:

(4) $$Z_{CPE}=\frac{1}{Y_{0}}{\left(j\omega \right)}^{-n}$$

where j represents the imaginary unit, which is equal to −1^(1/2)^, and ω indicates the angular frequency. Y_0_ in this equation represents the capacitance of the electrochemical corrosion testing system, and *n* typically ranges from 0.5 to 1. Table 2 lists parameters such as R_s_, R_p_, *n*, and Y_0_. The findings indicate that the *n* values for all composite titanium samples are quite similar, suggesting that the dissolution surfaces are comparable under mixing conditions. Regarding R_p_, the higher the R_p_ value, the more difficult the charge transfer through the passivation film. The TCCU-70 alloy exhibited the highest R_p_ value, while the lowest was found in the same alloy, indicating that TCCU-70 possesses the best corrosion resistance, consistent with the polarization test results.

**Figure 9:**
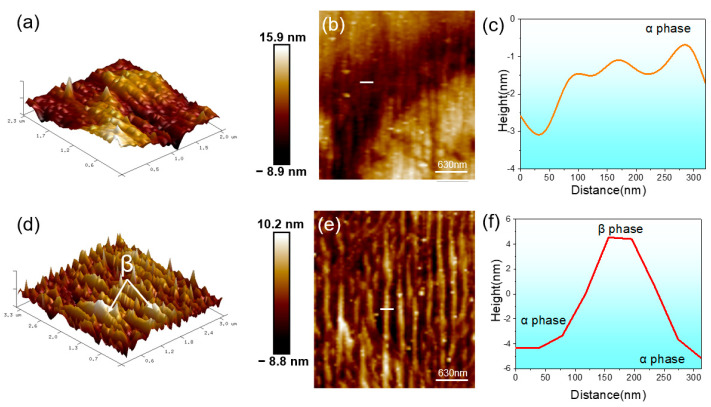
AFM surface morphology after the TCCU-70 immersed in 0.9% NaCl solution for 7 days: (**a**) 3D morphology of Ti-5Cu region; (**b**) 3D morphology of Ti6Al4V region; (**c**) micro-morphology of Ti-5Cu region; (**d**) micro-morphology of Ti6Al4V region; height distribution of (**e**) Ti-5Cu region and (**f**) Ti6Al4V region.

AFM was employed to examine the surface morphologies of the Ti6Al4V and Ti-5Cu regions of the TCCU-70 alloy after immersion in a 0.9 wt.% NaCl solution for 7 days to study the dissolution behavior of the Ti6Al4V-Ti-5Cu composite alloy, as shown in Figure 9. The findings revealed a uniform morphology in the Ti-5Cu zone (Figure 9a,c), whereas a hill-like morphology was observed in the Ti6Al4V region (Figure 9b,d). In the Ti6Al4V area, the valley-like phase was associated with the α phase, while the ridge-like phase corresponded to the β phase, as confirmed by the height distribution showing that the β phase exhibited a higher height value than the α phase (Figure 9e,f). It is noteworthy that the height distribution value in the Ti-5Cu region was lower than that in the Ti6Al4V region, possibly due to preferential dissolution occurring in the Ti-5Cu region compared to the Ti6Al4V region. Nevertheless, the AFM analysis was conducted at the nanoscale level, indicating minimal differences in dissolution between the Ti6Al4V and Ti-5Cu regions.

### 3.3. Antibacterial Property

**Figure 10:**
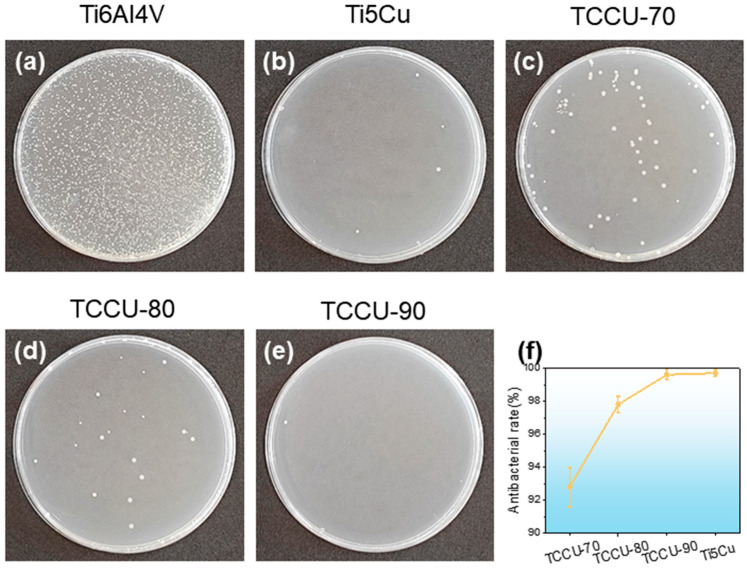
Typical bacteria colonies of alloys: (**a**) Ti6Al4V sample; (**b**) Ti-5Cu; (**c**) TCCU-70; (**d**) TCCU-80; (**e**) TCCU-90; and (**f**) antibacterial rates of alloys.

To assess the antibacterial properties of the Ti6Al4V-Ti5Cu alloy against Gram-negative bacteria, a co-culture experiment lasting 24 h was carried out, with results shown in Figure 10a–e. The Ti6Al4V alloy displayed a high concentration of bacterial colonies, whereas the Ti-5Cu showed significantly fewer. The TCCU-70, TCCU-80, and TCCU-90 samples had very limited bacterial colonization. A trend of decreasing bacterial colonies was observed as porosity increased, which aligns with previous microstructural findings. Increased porosity leads to a higher presence of the Ti_2_Cu phase in the alloy, which enhances its antibacterial properties. Figure 10f illustrates the antibacterial rates of the TCCU alloys. According to Standard GB/T 4789.2 [27], an antibacterial rate of R ≥ 90% signifies that the material has antibacterial properties. The TPMS structure shows an antibacterial rate exceeding 90%, while TCCU-90 reaches an impressive 99%, indicating strong antibacterial effectiveness.

## 4. Discussion

The electrochemical findings indicated that the presence of the interface did not notably impact the corrosion resistance of the Ti6Al4V-Ti5Cu composite alloy, with its interpenetrating structure, compared to the individual Ti6Al4V and Ti-5Cu alloys. Specifically, the Ti6Al4V-Ti5Cu composite alloy displayed a lower corrosion current density than both the Ti6Al4V and Ti-5Cu alloys separately. Additionally, the corrosion resistance of the Ti6Al4V-Ti5Cu composite alloy remained unaffected by the porosity of the TMPS structure. In this context, factors such as galvanic corrosion, phase, and precipitates likely contribute to the corrosion behavior observed in the Ti6Al4V-Ti5Cu composite alloy.

The primary concern in designing and manufacturing heterostructured metal composites lies in macro-galvanic corrosion, where the significant potential difference between different metals accelerates the dissolution of the anodic materials [28]. Within a galvanic couple, the metal with a lower potential, serving as the anode, undergoes more accelerated corrosion. The potential difference plays a pivotal role in determining the corrosion resistance of the composite—higher differences leading to increased galvanic corrosion tendencies. However, while the potential difference helps estimate the corrosion propensity in the metal composite, it alone cannot dictate the corrosion rate. This rate is influenced not only by the driving force but also by the polarization behavior, rendering the potential difference a gauge for estimating galvanic coupling tendencies rather than determining the corrosion rate [29]. In a galvanic couple, the surface state of the anodic component is generally dependent on the galvanic potential, following the principles of corrosion electrochemistry. Essentially, the passive film of the anodic component can dissolve when the galvanic potential falls within the active dissolution region. Conversely, a stable and compact passive film forms when the galvanic potential is in the passivation region, where the passive current equals the galvanic current of the anodic component. In this scenario, the anodic component undergoes self-corrosion rather than the accelerated galvanic corrosion effect [30]. Theoretically, the galvanic corrosion would occur when the potential difference is more than 60 mV [31]. The polarization curve analysis of Ti6Al4V and TiCu indicated a potential difference of 55 mV between them, falling within the passivation region (as illustrated in Figure 7b). This finding sets a prerequisite for averting macro-galvanic corrosion at the Ti6Al4V and Ti-5Cu interface. Several studies have consistently shown a minimal galvanic corrosion effect between titanium-based alloys and other differing alloys, even though the exact reasons behind this observation remain unclear [32,33]. For example, Zhao et al. [32], studying the galvanic corrosion between titanium (Ti60) and Zn alloy (H62), found the galvanic corrosion to be negligible despite a significant 360 mV potential difference. Given this analysis, it is reasonable to consider the galvanic corrosion effect between the Ti6Al4V and Ti-5Cu zones as negligible.

Additionally, a strong metallurgical bond at the interface also significantly contributes to minimizing or preventing macro-galvanic corrosion. Typically, the presence of coarse-sized precipitates may induce micro-galvanic corrosion between these precipitates and the matrix [34]. Higher densities of such precipitates embedded in the matrix can create more localized sites for corrosion attack, accelerating anodic dissolution [35,36,37]. Various studies have shown that multiple corrosion pits can form at the interface between heterostructured metals, especially worsening when coarse precipitates exist at this interface. This scenario can lead to preferential dissolution of either the matrix or the precipitates [38]. In the case of Cu-bearing titanium alloys, micro-galvanic cells can form due to the heterogeneous presence of Ti_2_Cu and the matrix, where Ti_2_Cu, being more noble than the Ti matrix, acts as the anode [39]. The presence of the Ti_2_Cu phase in a heterogeneous state might unfavorably impact the formation of a robust and dense passive film [40]. This phase can influence the stability of the passive film by forming a galvanic couple between the α phase and Ti_2_Cu phases, potentially resulting in the creation of a more irregular passive film [15]. From the observation in Figure 4, the nanoscale distribution of the Ti_2_Cu phase was uniform within the matrix, without any evidence of precipitation at the interface between the Ti6Al4V and Ti-5Cu zones. This uniform distribution of the Ti_2_Cu phase, along with the high-quality metallurgical interface, is believed to inherently lower the susceptibility of the Ti6Al4V-TiCu composite alloy to micro-galvanic corrosion. Therefore, these findings substantiate why the corrosion resistance of the Ti6Al4V-Ti5Cu composite alloy with an interpenetrating structure did not notably decrease in comparison to the Ti6Al4V-Ti5Cu composite.

In this study, we introduce an approach to craft a titanium composite alloy with a bioinspired architecture. However, certain limitations are evident. Our approach has not investigated the strength and ductility compared to the Ti6Al4V and Ti-5Cu alloy. Exploring biocompatibility and antibacterial properties remains crucial. Specially, elucidating the underlying antibacterial mechanisms is paramount. Our future research will concentrate on the Cu release-killing and contact killing mechanisms. Of particular interest is the investigation of contact killing mechanisms, arising from electron transport between the matrix and bacteria. We hypothesize that the Ti6Al4V and Ti-5Cu zones could facilitate electron transport channels between the matrix and bacteria, potentially leading to the anticipated antibacterial properties.

## 5. Conclusions

In this study, a novel Ti6Al4V-Ti5Cu metal composite with an interpenetrating structure was fabricated using a combination of SLM and SPS technology. The primary aim was to study the influence of heterogeneous materials on corrosion properties. Within the scope of this research, the key findings can be summarized as follows.

The SPS processing successfully established a strong metallurgical bond without observable defects like pores, micro-cracks, or precipitates at the interface between Ti-5Cu and the Ti6Al4V zones. The microstructure of both zones primarily comprised the laminar α phase, with distributed Ti_2_Cu precipitates in the Ti-5Cu zone.

The presence of the interface had no significant impact on the corrosion resistance of the Ti6Al4V-Ti5Cu composite alloy with an interpenetrating structure compared to individual Ti6Al4V and Ti-5Cu alloys. Specifically, the composite alloy exhibited a lower corrosion current density than both separate Ti6Al4V and Ti-5Cu alloys. This improved corrosion resistance can be attributed to the robust metallurgical bond at the interface. The antibacterial rate of the TPMS structure exceeds 90%, and that of TCCU-90 reaches as high as 99%, manifesting robust antibacterial activity.

## Figures and Tables

**Table 1:** The I_corr_, E_corr_, corrosion rate, and E_b_ value obtained from polarization curves.

Samples	E_corr_ (V/SCE)	I_corr_ × 10^−7^ (A·cm^−2^)	Corrosion Rate (mpy)
Ti6Al4V	−0.31 ± 0.02	2.41 ± 0.21	0.069 ± 0.037
Ti-5Cu	−0.34 ± 0.01	1.93 ± 0.37	0.082 ± 0.011
TCCU-70	−0.38 ± 0.03	1.58 ± 0.74	0.039 ± 0.063
TCCU-80	−0.35 ± 0.02	1.69 ± 0.38	0.067 ± 0.007
TCCU-90	−0.28 ± 0.04	1.83 ± 0.63	0.073 ± 0.005

**Table 2:** Equivalent circuit parameters calculated by fitting EIS data.

Samples	R_s_ (Ω·cm^2^)	R_p_ (kΩ·cm^2^)	Y_0_ (μQ^−1^·sn·cm^−2^)	*n*
TC4	22.83 ± 2.37	242.16 ± 59.56	30.25 ± 3.70	0.915 ± 0.031
Ti-5Cu	17.15 ± 2.68	232.72 ± 68.74	28.87 ± 2.59	0.824 ± 0.014
TCCU-70	27.12 ± 1.94	298.45 ± 52.48	27.01 ± 2.81	0.941 ± 0.025
TCCU-80	22.65 ± 2.43	254.25 ± 48.65	26.79 ± 3.62	0.929 ± 0.017
TCCU-90	19.76 ± 1.87	234.19 ± 43.62	32.64 ± 3.18	0.929 ± 0.042

## Data Availability

The original contributions presented in this study are included in the article. Further inquiries can be directed to the corresponding authors.

## References

[1] Geetha M., Singh A.K., Asokamani R., Gogia A.K. (2009). Ti based biomaterials, the ultimate choice for orthopaedic implants—A review. Prog. Mater. Sci..

[2] Yuan Z., He Y., Lin C., Liu P., Cai K. (2021). Antibacterial surface design of biomedical titanium materials for orthopedic applications. J. Mater. Sci. Technol..

[3] Wang P., Yuan Y., Xu K., Zhong H., Yang Y., Jin S., Yang K., Qi X. (2021). Biological applications of copper-containing materials. Bioact. Mater..

[4] (2024). Standard Specification for Unalloyed Titanium, for Surgical Implant Application.

[5] Zhang E., Zhao X., Hu J., Wang R., Fu S., Qin G. (2021). Antibacterial metals and alloys for potential biomedical implants. Bioact. Mater..

[6] Filipovic U., Dahmane R.G., Ghannouchi S., Zore A., Bohinc K. (2020). Bacterial adhesion on orthopedic implants. Adv. Colloid. Interface Sci..

[7] Jia M., Jin W., Li N., Lyu C., Wang Y. (2017). Related factors analysis and prevention of surgical implant infections in orthopedic patients. Chin. J. Nosocomiology.

[8] Pfang B.G., Garcia-Canete J., Garcia-Lasheras J., Blanco A., Aunon A., Parron-Cambero R., Macias-Valcayo A., Esteban J. (2019). Orthopedic Implant-Associated Infection by Multidrug Resistant Enterobacteriaceae. J. Clin. Med..

[9] Grischke J., Eberhard J., Stiesch M. (2016). Antimicrobial dental implant functionalization strategies—A systematic review. Dent. Mater. J..

[10] Liu J., Li F., Liu C., Wang H., Ren B., Yang K., Zhang E. (2014). Effect of Cu content on the antibacterial activity of titanium-copper sintered alloys. Mater. Sci. Eng. C Mater. Biol. Appl..

[11] Zhang E., Li F., Wang H., Liu J., Wang C., Li M., Yang K. (2013). A new antibacterial titanium-copper sintered alloy: Preparation and antibacterial property. Mater. Sci. Eng. C Mater. Biol. Appl..

[12] Lei Z., Zhang H., Zhang E., You J., Ma X., Bai X. (2018). Antibacterial activities and biocompatibilities of Ti-Ag alloys prepared by spark plasma sintering and acid etching. Mater. Sci. Eng. C.

[13] Peng C., Zhang S., Sun Z., Ren L., Yang K. (2018). Effect of annealing temperature on mechanical and antibacterial properties of Cu-bearing titanium alloy and its preliminary study of antibacterial mechanism. Mater. Sci. Eng. C Mater. Biol. Appl..

[14] Ma Z., Ren L., Liu R., Yang K., Zhang Y., Liao Z., Liu W., Qi M., Misra R.D.K. (2015). Effect of Heat Treatment on Cu Distribution, Antibacterial Performance and Cytotoxicity of Ti–6Al–4V–5Cu Alloy. J. Mater. Sci. Technol..

[15] Zhang E., Wang X., Chen M., Hou B. (2016). Effect of the existing form of Cu element on the mechanical properties, bio-corrosion and antibacterial properties of Ti-Cu alloys for biomedical application. Mater. Sci. Eng. C Mater. Biol. Appl..

[16] Liu X.C., Liu Z., Liu Y.J., Zafar Z., Lu Y.J., Wu X., Jiang Y., Xu Z.G., Guo Z.H., Li S.J. (2022). Achieving high strength and toughness by engineering 3D artificial nacre-like structures inTi6Al4V-Ti metallic composite. Compos. Pt. B Eng..

[17] Lu Y.J., Liu X.C., Liu Y.J., Wu X., Jiang Y., Liu Z., Lin J.X., Zhang L.C. (2023). Corrosion behavior of novel titanium-based composite with engineering 3D artificial nacre-like structures. Compos. Part A Appl. Sci. Manuf..

[18] Fang Z.G.Z., Paramore J.D., Sun P., Chandran K.S.R., Zhang Y., Xia Y., Cao F., Koopman M., Free M. (2018). Powder metallurgy of titanium—Past, present, and future. Int. Mater. Rev..

[19] Yamanoglu R. (2019). Pressureless Spark Plasma Sintering: A Perspective from Conventional Sintering to Accelerated Sintering Without Pressure. Powder Metall. Met. Ceram..

[20] Eriksson M., Shen Z., Nygren M. (2005). Fast densification and deformation of titanium powder. Powder Metall..

[21] Crosby K., Shaw L.L., Estournes C., Chevallier G., Fliflet A.W., Imam M.A. (2014). Enhancement in Ti-6Al-4V sintering via nanostructured powder and spark plasma sintering. Powder Metall..

[22] Long Y., Zhang H., Wang T., Huang X., Li Y., Wu J., Chen H. (2013). High-strength Ti–6Al–4V with ultrafine-grained structure fabricated by high energy ball milling and spark plasma sintering. Mater. Sci. Eng. A.

[23] Muthuchamy A., Patel P., Rajadurai M., Chaurisiya J.K., Annamalai A.R. (2018). Influence of sintering temperature on mechanical properties of spark plasma sintered pre-alloyed Ti-6Al-4 V powder. Mater. Werkst. Bauteile Forsch. Pruf. Anwend..

[24] Fan Z., Huang G., Lu Y., Chen Y., Zeng F., Lin J. (2022). Full compression response of FG-based scaffolds with varying porosity via an effective numerical scheme. Int. J. Mech. Sci..

[25] (2004). Implants for surgery—Measurements of open-circuit potential to assess corrosion behaviour of metallic implantable materials and medical devices over extended time periods.

[26] (2003). Experimental Method of Antibacterial Property and Antibacterial Effect.

[27] (2022). Total Colony Measurement.

[28] Wei L., Qin W. (2022). Corrosion mechanism of eutectic high-entropy alloy induced by micro-galvanic corrosion in sulfuric acid solution. Corros. Sci. J. Environ. Degrad. Mater. Its Control.

[29] Hu S., Liu R., Liu L., Cui Y., Oguzie E.E., Wang F. (2020). Effect of hydrostatic pressure on the galvanic corrosion of 90/10 Cu-Ni alloy coupled to Ti6Al4V alloy. Corros. Sci..

[30] Dong K., Song Y., Chang F., Han E.-H. (2023). Galvanic corrosion mechanism of Ti-Al coupling: The impact of passive films on the coupling effect. Electrochim. Acta.

[31] Zhang X.G. (2011). Galvanic Corrosion. Uhlig’s Corrosion Handbook.

[32] Zhao P., Song Y., Dong K., Shan D., Han E.H. (2019). Effect of passive film on the galvanic corrosion of titanium alloy Ti60 coupled to copper alloy H62. Mater. Corros..

[33] Grosgogeat B., Reclaru L., Lissac M., Dalard F. (1999). Measurement and evaluation of galvanic corrosion between titanium/Ti6A14V implants and dental alloys by electrochemical techniques and auger spectrometry. Biomaterials.

[34] Lee C., Kang C.S., Shin K.S. (2000). Effect of galvanic corrosion between precipitate and matrix on corrosion behavior of As-cast magnesium-aluminum alloys. Met. Mater. Int..

[35] Lu Y., Zhang Z., Liu Y., Yu C., Zhang X., Liu X. (2024). Improving mechanical properties and corrosion behavior of biomedical Ti-3Zr-2Sn-3Mo-25Nb alloy through laser surface remelting. Surf. Coat. Technol..

[36] Gan Y.M., Zhou M.H., Ji C., Huang G.H., Chen Y., Li L., Huang T.T., Lu Y.J., Lin J.X. (2023). Tailoring the tribology property and corrosion resistance of selective laser melted CoCrMo alloys by varying copper content. Mater. Des..

[37] Li L., Chen Y.X., Lu Y.J., Qin S.J., Huang G.H., Huang T.T., Lin J.X. (2021). Effect of heat treatment on the corrosion resistance of selective laser melted Ti6Al4V3Cu alloy. J. Mater. Res. Technol. JMRT.

[38] Grimm M., Lohmüller A., Singer R.F., Virtanen S. (2019). Influence of the microstructure on the corrosion behaviour of cast Mg-Al alloys. Corros. Sci..

[39] Osório W.R., Cremasco A., Andrade P.N., Garcia A., Caram R.J.E.A. (2010). Electrochemical behavior of centrifuged cast and heat treated Ti–Cu alloys for medical applications. Electrochim. Acta.

[40] Wang J., Zhang S., Sun Z., Wang H., Ren L., Yang K. (2019). Optimization of mechanical property, antibacterial property and corrosion resistance of Ti-Cu alloy for dental implant. J. Mater. Sci. Technol..

